# Beyond translation: Engaging with culturally and linguistically diverse consumers

**DOI:** 10.1111/hex.12984

**Published:** 2019-10-18

**Authors:** Reema Harrison, Merrilyn Walton, Upma Chitkara, Elizabeth Manias, Ashfaq Chauhan, Monika Latanik, Desiree Leone

**Affiliations:** ^1^ School of Public Health and Community Medicine University of New South Wales Sydney NSW Australia; ^2^ School of Public Health University of Sydney Sydney NSW Australia; ^3^ School of Nursing and Midwifery Deakin University Burwood Vic. Australia; ^4^ Melbourne School of Health Sciences The University of Melbourne Melbourne Australia; ^5^ Multicultural Health Western Sydney Local Health District Penrith NSW Australia

**Keywords:** consumer engagement, focus group research, health‐care quality, migrants, patient engagement

## Abstract

**Background:**

In the context of an effective consumer engagement framework, there is potential for health‐care delivery to be safer. Consumers from culturally and linguistically diverse (CALD) backgrounds may experience several barriers when trying to engage about their health care, and they are not acknowledged sufficiently in contemporary strategies to facilitate patient engagement.

**Methods:**

Four focus group discussions were facilitated by bilingual fieldworkers in Arabic, Mandarin, Turkish and Dari in a district of Sydney, Australia that has a high proportion of CALD consumers. Each group included 5‐7 health‐care consumers who, using a topic guide, discussed their experiences of barriers and facilitators when engaging with health‐care services in Australia. Thematic analysis was undertaken to identify, analyse and report patterns in the data.

**Results:**

In all, 24 consumers participated. Six inter‐related themes emerged: navigating the health system; seeking meaningful interpretation; understanding and managing expectations; respectful professional care; accessing services; and feeling unsafe.

**Conclusions:**

The incorporation of strategies such as professional interpreters and migrant health workers may go some way to addressing the needs of culturally or linguistically diverse consumers and facilitate communication, but do not sufficiently address the range of barriers to consumer engagement identified in this work. Understanding consumer experience in the context of the complex factors that may be associated with poor engagement and poor outcomes such as health literacy, cultural, educational and linguistic background, and health‐care setting or condition, may contribute to better understanding about how to deliver quality health care to these patients.

## 1 BACKGROUND

Patient engagement can be conceptualized on a spectrum from consultation relating to information exchange through to partnership, in which patients contribute to decision making and are recognized as a part of the health‐care team.^1^, ^2^ Today, patients are increasingly recognized as the only ‘constant’ element in any given health‐care interaction in which multiple care providers and services will participate. As such, patients are now considered co‐creators in their health care and are no longer simply recipients of care but active contributors who share decisions if they wish to or are given the opportunity.^3^, ^4^

Patient engagement frameworks are designed to increase the capacity of health‐care consumers in shared decisions about the planning and delivery of health care. This notion of patient engagement in policy development and implementation has gained traction over the last 20 years.^2^ An effective patient engagement framework has the potential to make health‐care delivery safer; to increase the mutual accountability of patients and providers; to allocate resources that centre on patient priorities; and to be more responsive and patient‐centred.^3^, ^5^ Consumer engagement has a significant presence in health policy internationally, reflected in the range of patient engagement and partnership initiatives, evident in health‐care settings worldwide.^4^, ^6^, ^7^

Patient engagement strategies commonly focus on enabling patients to be proactive during medical visits—to ask questions and raise concerns to address their goals.^8^, ^9^, ^10^ More recently, the concept of patient activation (which describes the knowledge, skills and confidence that a patient has regarding their health and health care) has also been associated with the degree to which patients engage in their care.^11^ Contemporary research and policy frameworks in this area fail to adequately acknowledge the experience of migrant consumers.

Consumers from diverse cultural or ethnic backgrounds may also experience additional barriers to engagement with health care.^12^ Research has focused on consumer experiences of negative events, highlighting ‘extreme powerlessness’ in the context of migrant health‐care encounters.^13^, ^14^ Evidence relating to patient engagement and knowledge of the implications for quality of care are lacking. Health‐care encounters between health providers and consumers from different cultural backgrounds are increasingly commonplace in the context of multicultural society. Enhanced understanding of the factors that facilitate encounters between providers and consumers from different backgrounds is therefore valuable.^15^, ^16^, ^17^

Culturally and linguistically diverse (CALD) is a term used primarily in Australian contexts to describe those who were born overseas, speak languages other than the official national languages and/or have lower proficiency of native or national languages, and/ or who have parents who were born overseas.^18^ The term is limited by its lack of inclusion of other types of diversity but is widely used with the intention of capturing diversity that is broader than language or birth. CALD consumers, including refugee populations, often experience lower quality health care.^19^, ^20^ Language barriers, lack of social support, lower health literacy, lower socio‐economic status, greater incidence of ill health and a sense of disempowerment are factors that heighten inequities for these populations and place CALD consumers at greater risk of adverse patient safety incidents.^19^, ^21^, ^22^, ^23^, ^24^

Strengthening consumer engagement amongst CALD populations is one approach to improving the quality and safety for this population, yet CALD consumer engagement remains under‐researched. Current approaches that emphasize ‘questioning’ health professionals and engaging through a range of verbal communication strategies may not be suitable in instances of limited language proficiency or be culturally appropriate.^22^ Understanding how CALD communities engage with health services and the barriers experienced is crucial knowledge for the development of evidenced‐based policy and practice. This study therefore aims to capture perceptions of CALD consumers from a range of backgrounds regarding the barriers to, and enablers of, engagement in health care through native language focus group inquiry.^25^

## 2 METHODS

### 2.1 Design

A cross‐sectional qualitative descriptive study was undertaken to enable the capture of experiences and perceptions of engagement with health care. The study is reported in accordance with the Consolidated Criteria for Reporting Qualitative Studies (COREQ) guidelines to promote complete and transparent reporting of this focus group research.^26^

### 2.2 Setting

Community health centres in one local public health district in Sydney were selected due to the large and diverse CALD population they served, with 35% of the population served identifying as CALD and 43% speaking a language other than English.^25^, ^26^, ^27^ This research was a collaboration with the Multicultural Health Services, which work in collaboration with clinical, non‐clinical and community partners and consumers to improve access to health services, empower diverse communities to actively participate in their health care and improve organizational capacity to respond to health needs of CALD consumers and populations. Major language groups were Arabic, Mandarin, Dari and Turkish. In collaboration with Western Sydney multicultural health‐care workers via the Multicultural Health Services, we invited participants from these four language groups to take part in this research.

### 2.3 Fieldworker training and topic guide

Bilingual fieldworkers recruited participants and conducted each of the focus groups. The audio recording was transcribed into English by an accredited translator for each focus group.^25^ One fieldworker was designated to each group from the outset. All fieldworkers had prior experience in conducting focus groups regarding patient health service use. Fieldworkers also had tertiary qualifications in health, or a health‐related discipline obtained in their country of origin, Australia or in both countries and worked as bilingual workers for Multicultural Health Services. Training was provided by the research team prior to the focus groups commencing. They were advised of the purpose of the study and their role in conducting the focus groups. The topic guide was developed based on the research team's patient experience work in Australia and internationally, and with input from the multicultural health team.^28^, ^29^, ^30^ The topic guide first asked group members to introduce themselves and their background. The facilitator then supported the group through iterative discussion around four areas: experiences of health care in Australia; the services accessed; experiences and perceptions of quality and safety; and finally of interactions with health‐care staff and services.

### 2.4 Recruitment

Study advertisements in the four languages and in English were posted in community health services in the district. The flyers were a useful starting point for introducing and talking about the study. Four bilingual fieldworkers (one from each of the four language groups) also circulated the study information throughout their community networks. Interested persons contacted the bilingual fieldworkers or the chief investigator (RH). This approach facilitated potential participants to ask questions about the study in their own language. This recruitment approach of engaging potential participants through bilingual fieldworkers, community channels and multicultural health‐care workers has been successfully used in previous work conducted with CALD consumers in Western Sydney.^25^

### 2.5 Data collection

Language‐specific focus groups examined the participants' experiences of engagement with health services and explored their views across a range of language and cultural backgrounds.^31^, ^32^ Homogenous groups with a common language were selected to facilitate a synergy of ideas from the participants in the group.^30^ We sought to recruit five to eight participants to each group.^30^

A 90‐minute focus group was conducted in each of the four targeted languages (Dari/Pashto, Turkish, Mandarin and Arabic) by bilingual fieldworkers within the local health district. Times and venues convenient for the participants were agreed upon. Using the same bilingual workers to recruit and conduct, the focus groups helped participants to develop trust and rapport, making them feel more comfortable with the research.^25^ CALD consumers are often not comfortable providing negative comments, which has been described as ‘the happy migrant effect’.^14^ Fieldworkers were themselves from CALD backgrounds and already had trusted relationships within their communities. This approach encouraged the participants to speak freely and openly regarding their experiences of the health care without fear or favour.

Participants were welcomed and introduced to members of the group and the fieldworker. Participants were then briefed about the study, how the focus groups would be conducted and were reassured about their privacy. Participants were informed they could withdraw at any time. Consent forms in English and in each language were signed.

Fieldworkers facilitated discussion using the topic guide to steer the discussion but to also explore interesting lines of inquiry and enable free expression of views.^25^ The topic guide progressed through experiences of health care in Australia, perceptions of the quality and safety of care and experiences of trying to engage in discussion with health‐care staff regarding treatment planning and processes. Participants were asked to discuss the facilitators and barriers experienced when trying to contribute to discussion and decision making about their care and wider service delivery. Comments, thoughts and feelings of individuals were explored rather than seeking to gain only a consensus view.^25^, ^30^ Translators also attended the focus groups, taking notes of the content of the discussion. The content of the dialogue was transcribed verbatim, but in instances in which this did not reflect the meaning documented in the fieldnotes, translators sought to represent the true meaning. The English transcript was provided to fieldworkers for checking to make sure the transcript was accurate and reflected the thoughts, views and opinions appropriately.

### 2.6 Analysis

Thematic analysis was undertaken manually to identify, analyse and report patterns in the qualitative data.^33^ Transcripts were independently read by two researchers (UC, RH) who, once familiar with the breadth and depth of content, undertook a focused line by line analysis. Themes were generated from the initial coding and then grouped under broader categories through discussion with a third researcher (RH). The categories were then labelled with reference to the raw data. Interpretations of the data were resolved through discussion at each stage in this process.

## 3 RESULTS

Each group consisted of 5‐7 participants aged 25‐90 years old: Arabic (6); Mandarin (6); Turkish (5); and Dari/Pashto (7). Participants had been in Australia for durations ranging from 3 months to 30 years, with the Arabic participants being the most recently arrived group and generally of a younger age range. Some participants within the Arabic group identified themselves as refugees.

**Figure 1:**
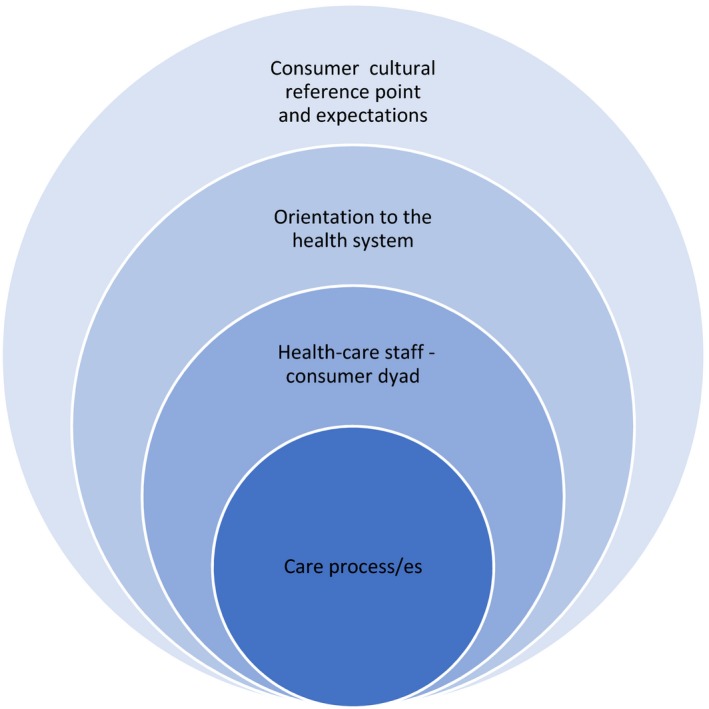
Levels of health‐care engagement at which barriers and facilitators identified

Our analysis revealed six inter‐related themes: navigating the health system; seeking meaningful interpretation; understanding and managing expectations; respectful professional care; access to services; and feeling unsafe. Figure 1 provides a representation of the points of interface with health care in which challenges and opportunities for engagement were identified.

### 3.1 Navigating the health system

Navigating the health system and identifying where to seek health care posed significant challenges to initial health‐care access, especially for emergency care. Persian and Arabic communities expressed confusion over what their course of action should be in the context of an urgent health condition requiring access to an emergency department. Similarly, participants expressed uncertainty regarding when and how to access services for existing conditions.

What I am concerned is for example, if I get shortness of breath and it is in the middle of the night, what should I do? Should I call ambulance? Should I seek help from neighbors or … in order to be able to get to the hospital. (Persian Group, P1)

I have an issue; I am pregnant, and I am given an appointment every 3 weeks. Assuming something serious happens to me within these 3 weeks who do I go to? Do I go back to the hospital that is very far from where I live or do, I call them or do I make an appointment. (Arabic Group, P3)

Patient‐education in language‐specific groups was identified as a useful approach to gain vital information regarding access to the appropriate point within the health‐care system. This view was expressed by all community groups, with several examples of the types of information that were identified through this process.

We didn't know until the foot health doctor came and conducted a program for us. We used to go to the GP and say that we had pain in our feet. But we didn't know another doctor was to take care of it. (Turkish Group, P4)

For example, when I needed to buy medications and I paid full price for them. I did not know that I could have waited until I received my healthcare card and that would have helped me. I would have paid 6 dollars instead of 40. I didn't know about this until I watched the seminar (Arabic Group, P6)

### 3.2 Seeking meaningful interpretation

Accurate and timely language interpretation was identified as central to facilitating engagement between patients and providers at any health‐care interface. Deficiencies in interpreting services were identified and acknowledged noting language barriers hard to navigate and overcome. Not only were interpreters absent in urgent health‐care encounters, such as those in emergency departments, they were also inconsistently available during booked appointments. This concern was voiced across all communities.

Yes, we did request one; but she didn't come and the specialist told us there is no interpreter. (Arabic Group, P3)

They say the children of the Turks help them, so we can reduce it (interpreting service).Yes, there aren't any left. (Turkish Group, P5)

In many public health facilities in Australia, professionally accredited interpreters are available via the Healthcare Interpreter Service in every public health facility and can be booked by health staff to support communication with consumers or other professionals. Non‐English‐speaking consumers can also request an interpreter, which will be organized by the health service at no additional cost. A ‘request’ for an interpreter was often identified as a factor that delayed access to care, with longer waiting time for appointments if an interpreter was requested. The inconvenience, additional expense and detriment to the health status of patients were described in each of the four groups.

My appointment was always postponed because it was very difficult to find an interpreter. (Mandarin‐speaking Group, P5)

…they again said they could not do that [extract a tooth that day]… there is not interpreter. I told them about my husband and that he could help with the language, but they said they could not do it in the absence of the hospital interpreter… finally I had to go to private dentist for extraction. (Persian Group, P2)

Arabic, Mandarin‐speaking and Persian communities described how interpreters did not attend the full consultation or help with pre‐ and post‐consultation needs such as booking further appointments. The community members described interpreters attending tightly packed appointments but noted this led to inefficiencies. Where interpreters only attended part of the consult, participants would come out without understanding the content and implications of the consultation.

We usually arrive before the interpreter and we wait until she comes and she usually arrives at the end of the appointment. Believe me, my husband had been going to these appointments for 2 years and sometimes we wait and the interpreter doesn't come at all and if they do come they are in a rush saying they have an appointment and they leave. (Arabic Group, P3)

Access to an interpreter was important, but participants also thought poor‐quality interpretation had an impact on the quality of the care process. Poor‐quality interpretation was a consistent issue identified amongst Persian and Arabic communities.

When an interpreter comes, and when we explain our problems, the interpreter relays something else. They cannot explain our problems appropriately and precisely. (Persian Group, P1)

My issue with the interpreter is that when I was in the hospital and I was asking the doctor questions the interpreter will omit a lot of things, sometimes it will be incorrect all together (Arabic Group, P4)

Participants described using strategies to overcome language barriers and the limitations of interpreter services, such as using staff members or social workers who know their language and communicating through children and family members. A common strategy used by the Mandarin‐speaking group was to seek out Mandarin‐speaking doctors and use health services known to them or their community.

We go to the social workers. For instance, we go to the (Turkish) Welfare (Association) to make an appointment. If you speak English, we come to you. We go to the neighbour's daughter, to the neighbour's child, to tell the truth (Turkish Group, P4)

There are many [Chinese doctors] in [xxx] hospital, easy to find. When they propose transferring to a farther place, I will ask whether there will be Chinese doctors. (Mandarin‐speaking Group, P5)

A highly valued strategy was the use of telephone applications to translate between the native and English language and facilitate direct communications with health professionals and support staff. One example of this is outlined below, but the use of apps and the benefits they afforded to participants was a feature common to all groups.

He [the doctor] needed to ask me questions and I didn't understand. So I used a translation app on my phone so that I can communicate with the doctor. That helped me a lot. (Arabic group, P2)

### 3.3 Understanding and managing expectations

Each focus group representing the four language communities identified a mismatch between their expectations of the availability and nature of health services, and the services and structures operating in the Australian health‐care system. This was a source of confusion and dissatisfaction with service delivery. But the quality of Australian health‐care services was highly regarded by participants; for example, Arabic community members valued the provision of services for older adult and child populations. Mandarin‐speaking community members also identified value in the online availability and transfer of medical results utilized in the Australian health‐care system.

Australian medical system is very good. For example, I go to see a doctor and get an examination. The results can be sent to the next doctor I see. (Mandarin‐speaking Group, P1)

A recent brain CT scan of my wife can be found in the online system, as well as the date of the film from last year. The comparison shows that there is no change. All this information can be found in the online system, which is not available in China. (Mandarin‐speaking Group, P1)

Nonetheless, many participants questioned the lack of affordable health care for the remainder of the population and reported that this was different to the systems in their country of origin.

For those that their ages range from 30‐50 years old, the health services available are less. For example, my tooth is hurting and I am confused why I have to pay for my treatment. (Arabic Group, P2)

Private health insurance was considered expensive and provided inadequate coverage; this issue was particularly voiced by Mandarin‐speaking respondents, reflecting the significant health insurance reforms that have taken place in China, but also by the Turkish group. Both groups provided several examples such as those below.

I took my husband to the eye doctor…[who] is going to put a lens… in his eye [the doctor] asked for 3,200 dollars. It (private cover) only covers half, not all of it, so we delayed it. (Turkish Group, P3)

The insurance premium increases every year. You still have to pay for physical therapy. I don't think it works for me. So I cancel it. (Mandarin‐speaking Group, P5)

Expectations regarding the role of general practitioners (GPs) were apparent. The limited role that GPs have in diagnosis and treatment was noted by many. Participants commonly identified lack of medical examination, high frequency of referral to a specialist and diagnostic test orders by GPs did not align with their expectations. They felt that GPs provided inadequate clinical assessment and did not use diagnostic skills such as physical examination or exercised clinical judgement to the degree they would expect.

When we have an illness and we go to GP, they advise to do a blood test. For everything, they say, do a blood test. They should examine first and then check whether a blood test is needed or not.. (Persian Group, P2)

The family doctor just gets in front of the computer, looks at the patient's face. He reads your illness from the computer. He doesn't ask you, he doesn't know…They don't even touch you. (Turkish Group, P5)

### 3.4 Respectful, professional care

Capacity to communicate was considered to be important, but being treated in a considerate, polite and friendly manner was equally important as a basis for consumer engagement. The value of respectful and professional behaviour in health‐care encounters was important in overcoming language barriers. Where health professionals were considered to be unfriendly or rude, patients described unmet needs.

She(nurse) didn't speak to me for two days because I was unable to learn her name… She would just look at me and go away… I asked her why she didn't speak to me. She said “you don't call my name. You keep forgetting it”. (Turkish Group, P4)

After delivery, the behaviour of nurses was very bad. They did not help …… I wanted to see a social‐ worker and this made them very upset… They thought I wanted to make a complaint, whereas my intention was to talk to them about my illnesses. Even when I asked to change the feeding bottle of the baby, they said; we know what to do and you don't need to tell us about that. (Persian Group, P1)

The [ultrasound] test was performed by an Indian woman whose attitude was horrible. She made me cry. She told me to take off my clothes and lie on the bed. I followed her instructions…she kept me lying there. However, she would not let me get up. The last test lasted more than half an hour. Then she took the report paper and left without telling me anything. I was still lying there. I was really cold. When I went out and tried to reason with her, she was extremely rude, suggesting that I should speak English if I want to see a doctor. (Mandarin‐speaking group, P5)

### 3.5 Access to services

All groups noted that inadequate and expensive health coverage resulted in poor access to health care. The high cost of medications and dental care were particular challenges, with implications for further health impacts when people did not access or discontinued their treatment. Confusion regarding the medications subsidised by the government on the Pharmaceutical Benefits Scheme was also apparent.

If you are sick, then you can go to GP without any cost, but buying medication is a serious issue…The government does not help with purchasing all types of medication. They only provide subsidy for medications which are for chronic illnesses. (Persian Group, P4)

Sometimes the medications prescribed by the GP is not bulkbilled and when I got buy it I get shocked that it costs 120 dollars and because I can't afford it I don't buy it…When I went to buy the medication he prescribed I found they were so expensive and I couldn't afford them. I just kept taking my medication that I had gotten with me from overseas (Arabic Group, P4)

Visa restrictions were identified as barriers to accessing health care due to the lack of availability of health benefits. Arabic and Persian communities were particularly affected by these issues.

I had toothache and it was so severe that I could not sleep. I went to see my GP and the GP said that I had to see a private dentist…I had so severe pain that I could not sleep for a month and I could not eat anything as well. I don't have support from Centrelink and therefore do not have health card. Centerlink does not give me healthcare card because the sub clause of my visa is 100 and they say I am not eligible. (Persian Group, P4)

### 3.6 Feeling unsafe

Concerns about the safety of care were apparent in the Persian, Mandarin‐speaking and Turkish groups. Many participants believed they or a family member had experienced an adverse event during health care and, in several cases, associated this with their cultural status.

The second operation was caused by a routine intestinal examination, which was highly recommended at that time…Her intestines were [damaged] during the examination. A while later, the director or someone from the hospital met with us and told us that they would take the responsibility. (Mandarin‐speaking Group, P1)

I had heart surgery in [xxx] in 1998. They took a vein from my leg. I see something shining in my leg. There was a piece of metal left there… A wire was left. But it was on my mind all the time. I would have pulled it out myself if I could. I couldn't. But what did he do? He pulled it out as if it was nothing. He just said sorry. (Turkish Group, P4)

Inability to communicate effectively with health professionals about their condition and safety concerns created significant anxiety across all communities, leaving them feeling unsafe during their treatment in hospital.

I had a heart attack when we came, but no doctor diagnosed it… It came out in the open when I came and was examined again by Turkish doctors….The main difficulty is the language. You are unable to explain yourself and the doctor doesn't understand you and there may be a wrong diagnosis, the wrong medication. We want it to be better and for them to be more careful. (Turkish Group, P4)

There were two nurses and I believe these two nurses were not experienced and even they could not work it how to put the needle [canula] in my hand for my medication…I was awake and could see them there and on the other hand I was alone …. As I was worried, then I asked them to call my home as I wanted someone from my family to be there…I could not communicate with the nurses because my English was not good and I did not have an interpreter….One problem was the language as I could not understand what they were telling and the other reason I felt unsafe was that when they put the needle in my hand, I got swelling on that spot and it was very painful and burning (Persian Group, P5)

Uncertainty regarding the skills, knowledge and supervision processes around junior and newly qualified doctors was of concern for Mandarin‐speaking community members, many of whom questioned how safe it was for interns to undertake surgical procedures in public hospitals. Some of the respondents reported feeling unsafe if they had to be treated by an intern.

When my wife underwent the examination of the intestine, the doctor in charge was said to be a chief physician, a good doctor. [How did he manage to damage] the intestines? Because it wasn't him who actually did it. The intestine was broken by an intern under his supervision. But it was still his responsibility. I didn't like this. Incompetent doctors should not be allowed to operate (Mandarin‐speaking Group, P1)

I received a letter saying that if I agreed to let an intern operate, surgery could be arranged right away. I refused the proposal as I didn't dare to let an intern operate on my eyes. I preferred waiting for the doctor who saw me. (Mandarin‐speaking Group, P5)

## 4 DISCUSSION

Our findings demonstrate that culturally and/or linguistically diverse consumers face several barriers to effective patient engagement at multiple levels of interface with health care, from primary to tertiary care settings. Strategies in place to address the needs of consumers, such as professional interpreters and migrant health workers, at times did facilitate communication, but do not sufficiently address the barriers to consumer engagement.

Language discordance is a well‐established barrier to communication, with implications for inhibiting opportunities for consumer engagement.^34^ Substandard and inadequate access to professional interpretation services by health professionals is recognized in existing literature.^35^ Inadequate interpretation is also a central barrier that went beyond the limited access to translators to facilitate questioning and understanding between professionals and consumers. Feeling that one was not interpreted correctly; that information was missed; and that the need for a translator impacted timely access to care contributed to a reduced sense of trust and quality of care provision amongst participants in this study. Family and carers feature centrally in patient engagement frameworks, yet opportunities for family members to contribute to and facilitate meaningful health‐care interactions were frequently missed in examples provided by participants.^36^ This is particularly important in the context of feeling unsafe, which emerged as a key theme.^12^

While participants said the ability to communicate with health‐care staff is an important basis for engagement, they also thought that non‐verbal communication, particularly physical actions, has the potential to disempower patients. A number of respondents described how, in the professional‐patient interaction, they were ignored or dismissed or the provider was confrontational rather than responsive to their concern. Being empowered as a health‐care consumer, with the skills and *opportunity* to contribute, is a fundamental basis for active participation in health‐care encounters.^37^

Participants said that the ability to navigate the health‐care system was an essential skill. Understanding how the health system works and how to access and use it are critical features of health literacy; such understanding is fundamental knowledge if consumers are to be effectively engaged.^38^, ^39^ Being unable to find and use the right service was identified as a considerable barrier to engagement leading to anxiety and reduced confidence amongst CALD consumers. The indication that those with poorer health literacy found this detrimental to engagement is unsurprising given the interrelationship between poorer health literacy and reduced quality of health care.^40^

### 4.1 Implications

Achieving effective consumer engagement for the CALD population may require a suite of strategies that reflects the diverse range of CALD groups. The range and influence of levels of education, experience, language proficiency and nature of cultural background are not captured in current evidence. Nuanced approaches for engagement with the diversity of CALD consumers are lacking but are sufficiently important for further research into practice.

There are several implications for research, policy and practice. Research regarding the association between patient engagement and poor quality and safety outcomes for CALD populations are required to better understand the nature of the relationship. Exploration of the factors associated with poor engagement amongst specific CALD groups and the individuals within these would contribute to better understanding about how to deliver quality health care to the diverse CALD population. Experiences common to specific communities were indicated in our work, but we did not capture sufficient data from any given population to generalize to any given community. Further research with specific communities would enable services with particular communities to understand more about how best to approach engagement efforts. The opportunity to work with people from a range of cultural backgrounds is critical during education and training of health professionals, but also dedicated emphasis on skills and strategies to develop an understanding of the patient as an individual, including their cultural, ethnic, language and/or religious background as a part of this.^41^ Training around cultural competence and humility in the context of patient‐centred care is critical.^42^

Telephone applications (apps) that allow consumers to translate ‘in real time’ with health‐care staff were identified by participants in this study as helpful and were seen as an empowerment tool. However because the safety implications of using these apps are unknown, health professionals are advised not to use them for medical communications.^43^ Current NSW Health policy prohibits the use of non‐professional interpretation in health‐care settings due to the potential for misinterpretation or inaccurate information; this is a core factor in the limited use of such apps by health‐care staff.^43^ In Australia, general practitioners (GPs) and specialists (who provide services under the universal coverage system of Medicare) have free access to the national Translating and Interpreting Service (TIS).^44^ Through the TIS, doctors can access accredited interpreters (usually by phone), for only the cost of a phone call. Despite the availability of this service, interpreters are not widely used by GPs and specialists.^45^ The use of translation apps is the focus of current research in health services in Sydney, Australia. Notwithstanding potential risks associated with apps for clinical interactions, there may be value in examining the use of such technology to address other factors that arise as barriers to engagement for CALD consumers, for example to support enhanced health literacy. Policy frameworks that enable such exploration are therefore important. A health system approach that facilitates understanding between consumers and health‐care providers of the consumers expectations of care and how they wish to interact with their health‐care services is central to enhancing engagement amongst CALD consumers. Cultural competence amongst health‐care workers at all levels is critical as a basis for this.

Prohibitive health‐care costs were raised by CALD consumers at several points during the focus group discussions, particularly in relation to medications and dental services. Linked to cultural competence is the recognition by health‐care workers and policymakers of differences in health system financing between countries, and the implications for CALD communities and their care in Australia. An understanding of the potential different expectations of each community may enable health professionals to prepare CALD consumers for these differences and highlight opportunities for reimbursement of costs. Policymakers may also consider the public health impacts of current models of financing for CALD communities when costs prohibit optimal primary or preventive care.

### 4.2 Limitations

Several factors may have been influential in shaping the data collected and resultant themes including the fewer male than female participants, the collection of data from only one district of Sydney and the variation apparent between groups in the participants' age range and the duration of time spent in Australia. Despite analysis of data across multiple focus groups, there are implications in the potential transferability of these findings due to the diversity amongst CALD populations with regard to factors such as time in Australia, English proficiency and the type of health problems experienced. Furthermore, it is not possible to readily determine from these data the challenges that are specific to cultural and linguistic diversity. The use of fieldworkers who were known to and trusted by the community was important to encourage open discussion but may have also influenced the nature of responses captured; increased comfort with the fieldworker led to more negative experiences being raised. The inclusion of only four language groups may also limit the transferability of our findings.

## 5 CONCLUSION

There is little evidence regarding the features of care that encourage effective patient engagement for patients from diverse cultural and linguistic backgrounds. Our findings indicate that there is some evidence to suggest that professional interpreters and migrant health workers can facilitate communication, but the provision of such services does not sufficiently address the range of barriers to consumer engagement that we report in this work. Understanding consumer experience in the context of the wider factors that may be associated with poor engagement and poor health outcomes such as health literacy, cultural, educational and linguistic background, and health‐care setting or condition is important. This approach may facilitate understanding about the approaches and circumstances in which opportunities for engagement and effective engagement arise with each CALD consumer.

## ETHICAL APPROVAL

The study received ethics approval from the Health Research Ethics Committee at the University of New South Wales Human Research Ethics Committee and the Western Sydney Local Health District.

## Data Availability

Access to the research data from this project may be discussed with the lead author and is subject to appropriate ethical approvals.
